# Pregnancy and neonatal outcomes of term deliveries of singleton pregnancies at different gestations in Sri Lanka: a multicentre prospective study

**DOI:** 10.1016/j.lansea.2025.100677

**Published:** 2025-10-03

**Authors:** Sachith Mettananda, Himali Herath, Ranod Madushith, Tiran Dias, Rasika Herath, Sampatha Goonewardena, Dhammica Rowel, Abner Elkan Daniel, Susie Perera

**Affiliations:** a Department of Paediatrics, University of Kelaniya, Kelaniya, Sri Lanka; b Colombo North Teaching Hospital, Ragama, Sri Lanka; c Perinatal Society of Sri Lanka, Colombo, Sri Lanka; d Family Health Bureau, Colombo, Sri Lanka; e Department of Obstetrics and Gynaecology, University of Kelaniya, Kelaniya, Sri Lanka; f Department of Community Medicine, Faculty of Medical Sciences, University of Sri Jayewardenepura, Nugegoda, Sri Lanka; g UNICEF Sri Lanka, Colombo, Sri Lanka; h Ministry of Health, Colombo, Sri Lanka

**Keywords:** Term gestation, Induction of labour, Caesarean section, Pregnancy outcome, Neonatal outcome

## Abstract

**Background:**

Delivery at ‘term’ is considered low risk for mothers and neonates. Evidence suggests variable outcomes at different gestations, even within ‘term’. This study aims to compare pregnancy characteristics and neonatal outcomes of delivery at different gestations at ‘term’.

**Methods:**

We analysed the data of the island-wide multicentre Sri Lanka Birth Weight Study, which recruited all live-born newborns in 13 hospitals over 2 months in 2023. Only data of singleton pregnancies and term neonates were included. Pregnancy complications and neonatal outcomes of each gestation were analysed by logistic regression.

**Findings:**

8053 ‘term’ singleton deliveries (1805, 2367, 2087, 1762, and 32 at 37, 38, 39, 40 and 41 weeks) were included. A higher proportion of mothers delivering at 37 weeks had pregestational diabetes (AOR: 7.84, 95% CI: 4.24–14.37), chronic hypertension (AOR: 4.37, 95% CI: 2.01–9.49), pregnancy-induced hypertension (AOR: 2.65, 95% CI: 1.92–3.66) and gestational diabetes (AOR: 1.96, 95% CI: 1.57–2.44) compared to mothers delivering at 39 weeks. The elective caesarean section rate was highest at 37 weeks (783, 43.4%) compared to 38 weeks (737, 31.1%) or higher gestations. Delivery at 37 weeks was associated with inferior neonatal outcomes of 5-min APGAR <8 (AOR: 3.04, 95% CI: 1.36–6.76), requiring resuscitation (AOR: 1.74, 95% CI: 1.27–2.38) and admission to intensive care (AOR: 1.62, 95% CI: 1.09–2.41) compared to 38 weeks.

**Interpretation:**

Neonates born at 38 weeks showed better outcomes than those born at 37 weeks. When elective delivery is necessary, postponing it from 37 weeks to at least 38 weeks would positively impact neonatal outcomes.

**Funding:**

UNICEF, Sri Lanka, funded the Sri Lanka Birth Weight Study. No funding obtained for this manuscript.

**Research in context**

**Evidence before this study**

The period between 37 and 41 weeks of gestation, conventionally classified as the ‘term’, is often perceived as a low-risk phase for both the mother and the neonate. Although many studies have described the outcome of pregnancies before 37 weeks, only a few studies have evaluated the outcome of deliveries happening at different gestations within term. Even the studies performed are conducted in developed, high-income countries and have compared early-term (37 + 0 to 38 + 6 weeks) and late-term (≥39 weeks) neonates. We searched PubMed for peer-reviewed papers published from inception to 12/09/2025 using the terms ‘pregnancy outcome OR neonatal outcome AND 37 versus 38 weeks of gestation AND South Asia’. This search revealed only one study conducted in nine low- and middle-income countries across sub-Saharan Africa, South and East Asia, and Latin America. This study also compared outcomes of early-term (37 + 0 to 38 + 6 weeks) and late-term (39 + 0 to 41 + 6 weeks) as combined groups and did not evaluate the outcomes of deliveries at individual gestations. To our knowledge, there were no studies that compared pregnancy and neonatal outcomes of deliveries at different gestations at ‘term’ in developing countries in South and Southeast Asia.

**Added value of this study**

This study examined the pregnancy and neonatal outcomes of singleton deliveries at individual gestations within term in Sri Lanka, a low-middle-income country in South Asia. It was performed over 2 months as a part of a large country-wide cohort study of all live-born neonates in thirteen hospitals from all nine provinces in Sri Lanka. The study included 8053 ‘term’ singleton deliveries and data were collected between 12 and 24 h of birth by directly interviewing mothers and perusing patient records.

**Implications of all the available evidence**

Neonates born at 38 weeks of gestation showed better outcomes compared to neonates born at 37 weeks, with lower prevalence of low birth weight, perinatal asphyxia, resuscitation at birth, and admission to the neonatal intensive care unit. These findings indicate that when elective deliveries are indeed necessary, planning them at or after 38 weeks, as opposed to 37 weeks, would have a significant positive impact on neonatal outcomes, especially in low- and middle-income countries in South Asia.

## Introduction

The Sustainable Development Goals (SDGs) set by the United Nations aim to reduce global neonatal mortality to at least 12 per 1000 live births by 2030.^1^ The member countries are expected to work towards this goal by ending the preventable deaths of newborns.^2^ Sri Lanka has set an SDG target to reduce the neonatal mortality rate from 6.8 per 1000 live births in 2022 to 4 per 1000 live births by 2030.^3^^,^^4^ With the current rate of progress, it appears that Sri Lanka would need to expedite an accelerated plan to achieve this target by the end of the decade.

One important determinant of neonatal mortality is the period of gestation at birth of newborns.^5^^,^^6^ The risks and complications of newborns born preterm, before completion of 37 weeks, are well described.^7^ Preterm neonates are at a higher risk of many complications that include low birth weight, respiratory distress, hypotension, hypoglycaemia, seizures and jaundice.^8^

The period between 37 and 41 weeks of gestation, conventionally classified as the ‘term’, is often perceived as a low-risk phase for both the mother and the neonate.^9^ The characteristics and outcomes of newborns born at any gestation within ‘term’ are perceived not to be significantly different. However, as the ‘term’ period spans a full five weeks, there could be differences in the characteristics and outcome of neonates born at different gestations within ‘term’.^10^ These differences, albeit subtle, could have a considerable impact on the outcome of pregnancies, particularly in low- and middle-income countries (LMICs).

Although the pregnancy and neonatal outcomes of term and preterm deliveries are well-studied worldwide, the characteristics and outcomes of neonates born at different gestations within the ‘term’ are understudied. Also, the few studies that have evaluated the outcome among these babies have been done in developed countries and have compared the early-term (37 + 0 to 38 + 6 weeks) and late-term (≥39 weeks) neonates.^11^^,^^12^ Studies that evaluate the pregnancy characteristics and neonatal outcomes at individual gestation weeks at ‘term’ are rare. This study aims to compare the pregnancy characteristics and neonatal outcomes of babies born at different gestations–i.e., 37, 38, 39, 40 and 41 weeks–within ‘term’.

## Methods

### Study design and setting

This study was conducted as part of the Sri Lanka Birth Weight Study—a countrywide multicentre prospective study—carried out in Sri Lanka over 2 months from 01 August to 30 September 2023. Thirteen hospitals were selected to represent all nine provinces, all tiers of hospitals and 20% of births in the country during the study period.^13^

### Study population

All live newborns born in the selected hospitals during the study period were recruited consecutively between 12 and 24 hours of age for the Sri Lanka Birth Weight Study after obtaining informed written consent from mothers. Only the data of term neonates of singleton pregnancies delivered between 37 + 0 and 41 + 6 weeks of gestation were included in the present study.

### Data collection procedure

Data were collected using an interviewer-administered questionnaire by interviewing mothers and perusing patient records. The questionnaire contained questions on socio-demographic background, pre-pregnancy medical complications, obstetric complications, delivery details, gestational period, birth weight and immediate neonatal outcomes. Mode of delivery, whether the caesarean section (CS) is elective or emergency and indications for CSs were gathered from inward patient records as determined by the consultant obstetrician responsible for delivery care. CSs that were planned and scheduled in advance and performed without immediate maternal or fetal compromise were defined as elective CS, whereas CSs that were unplanned and performed due to an acute or unforeseen maternal or fetal indication were defined as emergency CS.

Birth weight was measured to the nearest 5 g by trained healthcare personnel attending the delivery. The gestational age was calculated using the working expected date of delivery determined by the treating obstetric team based on the last regular menstrual period and the dating ultrasound scan (when available). Pregnancies with uncertain dates were excluded.

### Statistical analysis

Data were analysed using IBM SPSS Statistics 29.0. Analyses were based on complete cases, and data imputation was not performed as there were only a few missing data points in key variables. Frequencies, percentages, means with standard deviations and medians with interquartile ranges were used to present descriptive statistics. The Independent sample t-test was used to compare means, and the Mann–Whitney U test was used to compare medians. The chi-square test was used to determine associations between categorical variables and gestational ages.

Logistic regression was used to determine the maternal age- and parity-adjusted odds ratios (AOR) when analysing associations between maternal medical or obstetric complications and gestational ages. Associations between neonatal complications and gestational ages were determined by logistic regression after adjusting for maternal age, parity, maternal medical complications (pregestational diabetes, chronic hypertension, cardiac diseases, hypothyroidism, asthma, epilepsy, and chronic kidney disease) and obstetric complications (pregnancy-induced hypertension, gestational diabetes, urinary tract infection, chorioamnionitis, placenta previa, and placental abruption). Logistic regression models were constructed by entering all the above confounding variables into the model and gestational ages pairwise. The 39-week gestation was considered as the reference category, as previous studies have shown that the rate of many adverse outcomes continues to decline until 39 weeks, and 39 weeks is the standard gestation for elective deliveries according to most guidelines.^14^^,^^15^ A similar subgroup analysis was performed among neonates delivered by elective caesarean section. All analyses, including subgroup analysis, were defined *a priori* to identify associations between pregnancy characteristics or neonatal outcomes and gestational ages.

### Ethical approval

Ethical approval was obtained from the Ethics Review Committee of the Sri Lanka College of Paediatricians (Ref. SLCP/ERC/2023/09). Administrative approval was obtained from the Director General of Health Services and the Directors of participating hospitals. Informed written consent was obtained from participating mothers before recruitment to the Sri Lanka Birth Weight Study.

### Role of the funding source

UNICEF, Sri Lanka, funded the Sri Lanka Birth Weight Study. No specific funding was obtained for this manuscript. The funding source was not involved in study design, data collection, analysis, interpretation of data, writing of the manuscript and the decision to submit the paper for publication.

## Results

A total of 8053 ‘term’ singleton deliveries were included, of which 1805 (22.4%), 2367 (29.4%), 2087 (25.9%), 1762 (21.9%) and 32 (0.4%) were delivered at 37, 38, 39, 40 and 41 completed weeks of gestation, respectively.

### Socio-demographic characteristics

The socio-demographic characteristics of mothers who delivered at each gestation at ‘term’ are shown in Supplemental Table S1. The mean ages of mothers who delivered their babies at 37, 38, 39, 40 and 41 weeks of gestation were 29.9 (SD ± 5.6), 29.1 (SD ± 5.3), 27.7 (SD ± 5.2), 27.7 (SD ± 5.1) and 27.0 (SD ± 4.0) years, respectively. A higher proportion of working mothers delivered at 37 and 38 weeks than at 39 and 40 weeks (Table 1). A higher proportion of mothers from high-income families delivered at earlier gestations at 37 and 38 weeks compared to 39–41 weeks.

**Table 1:** Associations between socio-demographic characteristics and deliveries at different gestations at term.

Characteristic	37-week gestation (N = 1805)	38-week gestation (N = 2367)	39-week gestation (N = 2087)	40-week gestation (N = 1762)	41-week gestation (N = 32)	p-value^a^
Mother's employment status^b^						
Working mother	427 (23.7%)	536 (22.7%)	412 (19.8%)	349 (19.8%)	7 (21.9%)	p = 0.008
Housewife	1374 (76.3%)	1828 (77.3%)	1671 (80.2%)	1411 (80.2%)	25 (78.1%)	
Father's occupation^c^						
Non-professional	1390 (77.7%)	1832 (78.0%)	1662 (80.1%)	1432 (82.0%)	24 (77.4%)	p = 0.007
Professional	400 (22.3%)	516 (22.0%)	412 (19.9%)	314 (18.0%)	7 (22.6%)	
Mother's education level^d^						
OL or lower	988 (54.7%)	1330 (56.2%)	1191 (57.1%)	1017 (57.1%)	20 (62.5%)	p = 0.379
AL or higher	817 (45.3%)	1037 (43.8%)	895 (42.9%)	745 (42.3%)	12 (37.5%)	
Father's education level^e^						
OL or lower	1098 (61.3%)	1468 (62.4%)	1348 (64.9%)	1119 (63.8%)	20 (62.5%)	p = 0.178
AL or higher	693 (38.7%)	885 (37.6%)	729 (35.1%)	634 (36.2%)	12 (37.5%)	
Monthly family income (LKR)^f^						
≤50,000	981 (55.8%)	1360 (58.8%)	1259 (61.7%)	1054 (62.2%)	22 (71.0%)	p < 0.001
>50,000	778 (44.2%)	952 (41.2%)	783 (38.3%)	640 (37.8%)	9 (29.0%)	

a p values were derived by Chi-square test.

b Missing data: 13 subjects.

c Missing data: 64 subjects.

d Missing data: 1 subject.

e Missing data: 47 subjects.

f Missing data: 215 subjects.

### Maternal medical complications

A higher proportion of mothers who delivered at 37 weeks had pregestational diabetes (AOR: 7.84, 95% CI: 4.24–14.37) and chronic hypertension (AOR: 4.37, 95% CI: 2.01–9.49) compared to mothers delivering at 39 weeks (Table 2). Similarly, a higher proportion of mothers who delivered at 38 weeks had pregestational diabetes compared to 39 weeks (AOR: 2.48, 95% CI: 1.29–4.77). The highest prevalence of preexisting cardiac diseases (41/2367, 1.7%) was seen among mothers in the 38-week group. Maternal hypothyroidism, asthma, epilepsy, and chronic kidney disease did not show associations with the gestational age at delivery. Similarly, the prevalence of maternal medical complications was not different between the mothers who delivered at 39 weeks and 40 weeks.

**Table 2:** Prevalence of maternal medical complications of deliveries at different gestations at term.

Maternal medical complication	Frequency (%) at different gestation categories					Adjusted odds ratios (AOR) and 95% CI referenced to 39-week gestation category		
	37-week gestation (N = 1805)	38-week gestation (N = 2367)	39-week gestation (N = 2087)	40-week gestation (N = 1762)	41-week gestation (N = 32)	AOR & 95% CI for 37-week gestation^a^	AOR & 95% CI for 38-week gestation^a^	AOR & 95% CI for 40-week gestation^a^
Pregestational diabetes	95 (5.3%)	41 (1.7%)	12 (0.6%)	5 (0.3%)	0	AOR: 7.84, CI: 4.24–14.37	AOR: 2.48, CI: 1.29–4.77	AOR: 0.49, CI: 0.17–1.4
Chronic hypertension	39 (2.2%)	23 (1.0%)	8 (0.4%)	2 (0.1%)	0	AOR: 4.37, CI: 2.01–9.49	AOR: 2.08, CI: 0.92–4.70	AOR: 0.29, CI:0.06–1.41
Cardiac diseases	20 (1.1%)	41 (1.7%)	21 (1.0%)	14 (0.8%)	0	AOR: 1.18, CI: 0.63–2.22	AOR: 1.82, CI: 1.07–3.10	AOR: 0.79, CI: 0.40–1.56
Hypothyroidism	76 (4.2%)	72 (3.0%)	69 (3.3%)	34 (1.9%)	0	AOR: 1.08 CI: 0.77–1.53	AOR: 0.83 CI: 0.59–1.17	AOR: 0.57 CI: 0.37–0.86
Asthma	128 (7.1%)	171 (7.2%)	141 (6.8%)	121 (6.9%)	2 (6.3%)	AOR: 1.01, CI: 0.79–1.31	AOR: 1.02, CI: 0.81–1.29	AOR: 1.02, CI: 0.79–1.31
Epilepsy	19 (1.1%)	21 (0.9%)	18 (0.9%)	13 (0.7%)	0	AOR: 1.45, CI: 0.75–2.80	AOR: 1.08, CI: 0.57–2.05	AOR: 0.85, CI: 0.41–1.75
Chronic kidney disease	4 (0.2%)	2 (0.1%)	3 (0.1%)	0	0	AOR: 1.81, CI: 0.39–8.35	AOR: 0.57, CI: 0.09–3.48	–

a AOR, Odds ratios adjusted for maternal age and parity in logistic regression.

### Obstetric complications

A higher proportion of mothers who delivered at 37 weeks had pregnancy-induced hypertension (AOR: 2.65, 95% CI: 1.92–3.66), gestational diabetes (AOR: 1.96, 95% CI: 1.57–2.44), and placenta previa (AOR: 13.23, 95% CI: 3.08–56.83) compared to mothers delivering at 39 weeks (Table 3). Urinary tract infection, chorioamnionitis, and placental abruption did not show associations with gestational age at delivery. The prevalence of antenatally detected fetal growth restriction (FGR) and oligohydramnios was higher among the mothers who delivered at 37 and 38 weeks compared to 39 weeks.

**Table 3:** Prevalence of obstetric complications of deliveries at different gestations at term.

Obstetric complication	Frequency (%) at different gestation categories					Adjusted odds ratios (AOR) and 95% CI referenced to 39-week gestation category		
	37-week gestation (N = 1805)	38-week gestation (N = 2367)	39-week gestation (N = 2087)	40-week gestation (N = 1762)	41-week gestation (N = 32)	AOR & 95% CI for 37-week gestation^a^	AOR & 95% CI for 38-week gestation^a^	AOR & 95% CI for 40-week gestation^a^
Pregnancy-induced hypertension	131 (7.3%)	86 (3.6%)	58 (2.8%)	26 (1.5%)	1 (3.1%)	AOR: 2.65, CI: 1.92–3.66	AOR: 1.32, CI: 0.94–1.87	AOR: 0.53, CI: 0.33–0.85
Gestational diabetes	252 (14.0%)	309 (13.1%)	145 (6.9%)	54 (3.1%)	0	AOR: 1.96, CI: 1.57–2.44	AOR: 1.94, CI: 1.57–2.39	AOR: 0.42, CI: 0.30–0.58
Urinary tract infection	81 (4.5%)	80 (3.4%)	94 (4.5%)	61 (3.5%)	0	AOR: 1.06, CI: 0.77–1.44	AOR: 0.76, CI: 0.56–1.04	AOR: 0.75, CI: 0.54–1.05
Chorioamnionitis	6 (0.3%)	4 (0.2%)	5 (0.2%)	6 (0.3%)	0	AOR: 1.43, CI: 0.42–4.84	AOR: 0.68, CI: 0.18–2.57	AOR: 1.40, CI: 0.42–4.60
Placenta previa	23 (1.3%)	12 (0.5%)	2 (0.1%)	4 (0.2%)	0	AOR: 13.23, CI: 3.08–56.83	AOR: 4.66, CI: 1.03–21.07	AOR:2.40, CI: 0.44–13.17
Placental abruption	5 (0.3%)	5 (0.2%)	3 (0.1%)	4 (0.2%)	0	AOR: 2.74, CI: 0.51–14.71	AOR: 2.45, CI: 0.47–12.79	AOR: 2.42, CI: 0.44–13.25
Fetal growth restriction	177 (9.8%)	114 (4.8%)	34 (1.6%)	14 (0.8%)	2 (6.3%)	AOR: 7.80, CI: 5.34–11.40	AOR: 3.28, CI: 2.22–4.85	AOR: 0.47, CI: 0.25–0.89
Oligohydramnios	89 (4.9%)	66 (2.8%)	34 (1.6%)	17 (1.0%)	2 (6.3%)	AOR: 3.77, CI: 2.51–5.68	AOR: 1.91, CI: 1.25–2.91	AOR: 0.58, CI: 0.32–1.04

a AOR, Odds ratios adjusted for maternal age and parity in logistic regression.

### Delivery characteristics

A higher proportion of primipara mothers delivered after 39 weeks compared to earlier gestations (Table 4). The proportion of pregnancies confined by caesarean section (CS) was higher at 37 weeks (1105/1805, 61.2%) compared to 38 (1117/2367, 47.2%), 39 (511/2087, 24.5%) and 40 (472/1762, 26.8%) weeks. Specifically, the elective CS rate was highest at 37 weeks (783/1805, 43.4%) compared to 38 (737/2367, 31.1%), 39 (127/2087, 6.1%), 40 (66/1762, 3.7%) and 41 (3/32, 9.4%) weeks. In fact, 783/1716 (45.6%) elective CSs done at term were performed at 37 weeks.

**Table 4:** Delivery characteristics of deliveries at different gestations at term.

Delivery characteristic	37-week gestation (N = 1805)	38-week gestation (N = 2367)	39-week gestation (N = 2087)	40-week gestation (N = 1762)	41-week gestation (N = 32)	p-value^a^
Parity^b^						
Primipara	664 (36.8%)	969 (40.9%)	1064 (51.0%)	951 (54.0%)	23 (71.9%)	p < 0.001
Multipara	1141 (63.2%)	1398 (59.1%)	1022 (49.0%)	810 (46.0%)	9 (28.1%)	
Mode of delivery						
Vaginal delivery (VD)	700 (38.8%)	1250 (52.8%)	1576 (75.5%)	1290 (73.2%)	19 (59.4%)	p < 0.001
Caesarean section (CS)	1105 (61.2%)	1117 (47.2%)	511 (24.5%)	472 (26.8%)	13 (40.6%)	
Onset of labour in VD^c^						
Spontaneous VD	423 (61.4%)	851 (69.5%)	1182 (76.5%)	787 (62.3%)	6 (31.6%)	p < 0.001
Induced VD	266 (38.6%)	373 (30.5%)	363 (23.5%)	476 (37.7%)	13 (68.4%)	
Mode of delivery						
Normal VD	683 (37.8%)	1209 (51.1%)	1521 (72.9%)	1231 (69.9%)	19 (59.4%)	p < 0.001
Forceps delivery	10 (0.6%)	20 (0.8%)	23 (1.1%)	29 (1.6%)	0	p = 0.018
Vacuum delivery	7 (0.4%)	21 (0.9%)	32 (1.5%)	30 (1.7%)	0	p < 0.001
Elective CS	783 (43.4%)	737 (31.1%)	127 (6.1%)	66 (3.7%)	3 (9.4%)	p < 0.001
Emergency CS	322 (17.8%)	380 (16.1%)	384 (18.4%)	406 (23.0%)	10 (31.3%)	p < 0.001

a p values were derived by Chi-square test.

b Missing data: 2 subjects.

c Missing data: 95 subjects.

Further analysis of vaginal deliveries revealed that 509/956 (53.2%) vaginal deliveries of mothers with medical or obstetric complications were induced deliveries, whereas 982/3878 (25.9%) vaginal deliveries of mothers with uncomplicated pregnancies were induced (Supplemental Table S2). 170/509 (33.3%) of induced vaginal deliveries of mothers with medical or obstetric complications were at 37 weeks. Even among the mothers with no medical or obstetric complications, 96/982 (9.7%) induced vaginal deliveries were performed at 37 weeks compared to later gestations.

Analysis of CS deliveries showed that 2254/6132 (36.8%) uncomplicated pregnancies were confined by CS, of which 1188 (52.7%) were elective CSs. Of the CSs of uncomplicated pregnancies, 481/1188 (40.4%) were performed at 37 weeks. The indications for CS at individual gestations are summarised in Supplemental Table S3.

### Neonatal outcome

Another important outcome of our study was the comparison of neonatal outcomes at different gestations. The mean birth weight of neonates born at 37 weeks (2730 ± SD426 g) was approximately 130–350 g lower than the mean birth weight of neonates born at 38 (2867 ± SD382 g), 39 (3012 ± SD377 g), or 40 (3080 ± SD353 g) weeks. This difference was seen in both genders (Supplemental Figure S1).

Low birth weight showed a stronger association with earlier gestations, with a higher proportion of neonates born at 37 weeks (529/1805, 29.4%) having low birth weight compared to babies born at 38 weeks (359/2367, 15.2%) or 39 weeks (154/2087, 7.4%) (Table 5). Nearly half (529/1122; 47.1%) of ‘term’ low birth weight babies were delivered at 37 weeks. None of the babies born at 38 or after 38 weeks had extreme (<1000 g) or very low birth weight (1000–1499 g). However, 4 (0.2%) neonates born at 37 weeks had very low birth weight. The highest rate of small for gestational age was observed among neonates born at 40 weeks (424/1762, 24.1%) and 41 weeks (14/32, 43.8%).

**Table 5:** Neonatal outcomes within first 24 h of life of neonates born at different gestations.

Neonatal outcome	Frequency (%) at different gestation categories					Adjusted odds ratios (AOR) and 95% CI referenced to 39-week gestation category		
	37-week gestation (N = 1805)	38-week gestation (N = 2367)	39-week gestation (N = 2087)	40-week gestation (N = 1762)	41-week gestation (N = 32)	AOR & 95% CI for 37-week gestation^a^	AOR & 95% CI for 38-week gestation^a^	AOR & 95% CI for 40-week gestation^a^
Low birth weight (<2500 g)^b^	529 (29.4%)	359 (15.2%)	154 (7.4%)	80 (4.5%)	0	AOR: 6.58, CI: 5.37–8.07	AOR: 2.47, CI: 2.02–3.03	AOR: 0.56, CI: 0.43–0.75
Small for gestational age^c^	338 (18.8%)	410 (17.3%)	379 (18.2%)	424 (24.1%)	14 (43.8%)	AOR:1.25, CI: 1.05–1.48	AOR: 1.03, CI: 0.88–1.20	AOR: 1.39, CI: 1.18–1.62
5-min APGAR <8^d^	20 (1.1%)	9 (0.4%)	12 (0.6%)	20 (1.1%)	0	AOR: 1.91, CI: 0.90–4.05	AOR: 0.58, CI: 0.24–1.41	AOR: 2.10 CI: 1.01–4.394
Resuscitation at birth	97 (5.4%)	76 (3.1%)	84 (4.0%)	74 (4.2%)	2 (6.3%)	AOR: 1.36, CI: 0.99–1.86	AOR: 0.80, CI: 0.58–1.11	AOR: 1.05, CI: 0.76–1.45
Death within first 24 h of life	2 (0.11%)	1 (0.042%)	1 (0.047%)	1 (0.056%)	0	AOR: 2.83, CI: 0.24–32.50	AOR: 1.02, CI: 0.06–17.07	AOR: 1.04, CI: 0.06–16.77
Admitted to the NICU	60 (3.3%)	47 (2.0%)	42 (2.0%)	50 (2.8%)	2 (6.3%)	AOR: 1.49, CI: 0.97–2.27	AOR: 0.90, CI: 0.59–1.39	AOR: 1.39, CI: 0.91–2.12

a AOR, Odds ratios adjusted for maternal age, parity, maternal medical and obstetric complications in logistic regression.

b Missing data: 4 subjects.

c Missing data: 5 subjects.

d Missing data: 1 subjects.

Neonates born at 38 weeks of gestation showed the best immediate neonatal outcome among all ‘term’ neonates, with lower proportions of them having 5-min APGAR <8 (9/2367, 0.4%), requiring resuscitation at birth (76/2367, 3.1%) and needing neonatal intensive care unit (NICU) admission (47/2367, 2.0%). Delivery at 37 weeks was associated with higher incidence of having 5-min APGAR <8 (AOR: 3.04, 95% CI: 1.36–6.76), requiring resuscitation at birth (AOR: 1.74, 95% CI: 1.27–2.38) and admission to the NICU (AOR: 1.62, 95% CI: 1.09–2.41) compared to birth at 38 weeks of gestation (Supplemental Table S4). There was no marked difference in adverse immediate neonatal outcomes between neonates born at 38 and 39 weeks of gestation.

The subgroup analysis of neonates delivered by elective CS confirmed the finding of better neonatal outcomes at 38 weeks. Elective CS at 37 weeks was strongly associated with a higher incidence of requiring resuscitation at birth (AOR: 2.64, 95% CI: 1.50–4.64) and admission to the NICU (AOR: 2.23, 95% CI: 1.14–4.36) compared to elective CS at 38 weeks of gestation (Supplemental Table S5).

## Discussion

In this study, we examined the pregnancy characteristics and neonatal outcomes of deliveries at individual gestations within ‘term’ in Sri Lanka, an LMIC in South Asia, to understand the impact of deliveries at different gestational ages within ‘term’. The study included over 8000 pregnancies and neonates born at ‘term’ from all nine provinces in Sri Lanka.

The study revealed that a large proportion (22.4%) of term deliveries occur at 37 weeks. This figure was higher than those reported from developing countries; the USA and the UK reported 11.6% and 10.8% deliveries in 37 weeks, respectively.^11^^,^^16^ The higher proportion of term deliveries at 37 weeks in this study was primarily contributed to by a high proportion of CS. Over 60% of deliveries at 37 weeks were by CS, and 43% were by elective CS. Nearly half (45.6%) of elective CS done at term were performed at 37 weeks, suggesting many obstetricians prefer to plan elective CS at 37 weeks rather than at later gestations. Even among mothers with uncomplicated pregnancies, over 40% of planned elective CS were performed at 37 weeks.

Our results show that over one-third (38%) of vaginal deliveries at 37 weeks were induced deliveries. This contrasts with the recent guidelines recommending planning elective CS and induced vaginal deliveries after 39 weeks.^14^^,^^15^^,^^17^ We believe that the overall perception of any gestation at term as low risk for the mother and neonate would have contributed to the practice of planning elective deliveries at 37 weeks.

Our study revealed that many maternal obstetric complications, including pregnancy-induced hypertension, gestational diabetes, placenta previa, and oligohydramnios, were higher among mothers who delivered at 37 weeks. This is consistent with previous studies, which reported a higher incidence of gestational hypertension, preeclampsia, and gestational diabetes at earlier gestations at term compared to late-term gestations.^11^

Another important observation in the study is that the birth weights of babies delivered at 38 and ≥ 39 weeks were higher than those delivered at 37 weeks. Mean birth weights increased by approximately 150 g per week from 37 to 38 and then to 39 weeks, indicating steady fetal weight gain even after reaching ‘term’. The fact that the fetus continues to grow beyond 37 weeks is well established and has been an important factor in advocating for continuing pregnancies until 39 weeks. Our results confirm that extending the pregnancy by 1–2 weeks, even after reaching term, could have a greater impact on reducing the prevalence of low birth weight.^13^

Another critical finding of the study is that delivery at 38 weeks of gestation was associated with better neonatal outcomes than birth at 37 weeks. At 38 weeks, a lower proportion of neonates had perinatal asphyxia and were resuscitated at birth and admitted to neonatal intensive care units. Similar findings were observed in the analysis of the elective CS subgroup, indicating better outcomes of neonates delivered by elective CS at 38 weeks compared to 37 weeks. These results were obtained through logistic regression analysis after adjusting for many confounding variables that affect neonatal outcomes; therefore, they are likely to represent the actual effect of the difference in delivery gestation. However, the impact of unaccounted residual confounders cannot be completely ruled out.

Overall, our findings show that if elective deliveries were to happen at 38 weeks rather than 37 weeks, wherever feasible, they would have superior neonatal outcomes. Nonetheless, this recommendation should be implemented with caution, as some obstetric complications like gestational diabetes and pregnancy-induced hypertension will necessitate elective delivery at earlier gestations.^18^

Our study did not evaluate several other advantages of continuing pregnancy beyond 37 weeks. The most notable advantage is the fetal brain development after 37 weeks. A population-based record linkage cohort study in Australia showed that planned delivery before 39 weeks was associated with increased risk of poor child development at school age.^19^

Additionally, there are economic advantages to postponing planned deliveries beyond 37 weeks. We have shown that neonates born at 37 weeks are more likely to be admitted to the NICU compared to later gestations. NICU admissions are costly and could significantly burden the already constrained health services in LMICs. Similar findings were reported in studies done in other countries, which showed that the hospital costs decrease with each week of gestational age until 39 weeks.^20^

One important limitation of our study is the lack of data on stillbirths. The Sri Lanka Birth Weight Study only recruited live-born infants; therefore, we could not evaluate the prevalence of pregnancy losses at each gestation. Uncertainty around determining the gestational age, a limitation common to neonatal studies, is also a limitation of this study. Reliance on the treating obstetrician's determination on whether the caesarean section is elective or emergency, and the indication for the caesarean section and potential inaccuracies in medical records are also limitations of our study. Lack of data on long-term neonatal outcomes is another limitation. The data was collected between 12 and 24 h of birth, and only neonatal outcomes of up to 24 h were recorded. However, it is less likely to significantly alter the overall conclusions of the study, as we have observed better outcomes in all aspects of neonatal health at 38 weeks compared to 37 weeks.

In conclusion, our study revealed that over one-fifth of term neonates were delivered at 37 weeks and over 40% of them were delivered by elective CS. Nearly half of the elective CS done at term were performed at 37 weeks. Neonates born at 38 weeks of gestation showed better outcomes compared to neonates born at 37 weeks, with a lower incidence of low birth weight, perinatal asphyxia, resuscitation at birth, and admission to NICU. These findings indicate that when elective delivery is indeed necessary, planning it at 38 weeks, as opposed to 37 weeks, would have a significant positive impact on neonatal outcome, especially in LMICs like Sri Lanka.

## Contributors

SM, HH, TD, RH and SP conceptualised the study. SM, RM, TD, and RH performed the literature review. SM, HH, SG, DR, AED and SP were involved in data collection. SM, RM, TD, and RH analysed data. SM, HH, and RM had access to raw data and verified the data. SM, RM, TD and RH wrote the manuscript. SM had final responsibility for the decision to submit for publication. All authors read and approved the final manuscript.

## Data sharing statement

Data will be available on a reasonable request to the corresponding author.

## Declaration of interests

None of the authors declare competing interests.
