# Supplementary material for: Pregnancy and neonatal outcomes of term deliveries of singleton pregnancies at different gestations in Sri Lanka: a multicentre prospective study
Source: Lancet Reg Health Southeast Asia. 2025 Oct 3;42:100677. doi: 10.1016/j.lansea.2025.100677 (PMC12524168; doi:10.1016/j.lansea.2025.100677)
Supplement: Supplemental Tables and Figures [file mmc1.pdf]

Manuscript Title: Pregnancy and neonatal outcomes of term deliveries of singleton pregnancies at different gestations in Sri Lanka: a multicentre prospective study

Supplemental Table 1 – Socio-demographic characteristics of mothers

Characteristic	***37-week gestation (N=1805)***	***38-week gestation (N=2367)***	***39-week gestation (N=2087)***	***40-week gestation (N=1762)***	***41-week gestation (N=32)***	***Total (N=8053)***
Maternal age[1]						
=<19 years	61 (3.4%)	80 (3.4%)	118 (5.7%)	82 (4.7%)	2 (6.3%)	343 (4.3%)
20-30 years	940 (52.1%)	1368 (57.8%)	1360 (65.2%)	1186 (67.3%)	25 (78.1%)	4879 (60.6%)
31-35 years	490 (27.1%)	640 (27.0%)	450 (21.6%)	352 (20.0%)	5 (15.6%)	1937 (24.1%)
>35 years	314 (17.4%)	279 (11.8%)	159 (7.6%)	141 (8.0%)	0	893 (11.1%)
Ethnicity						
Sinhala	1087 (60.2%)	1316 (55.6%)	1119 (53.6%)	795 (45.1%)	10 (31.3%)	4327 (53.7%)
Tamil	419 (23.2%)	634 (26.8%)	599 (28.7%)	577 (32.7%)	20 (62.5%)	2249 (27.9%)
Muslim	298 (16.5%)	413 (17.4%)	366 (17.5%)	389 (22.1%)	2 (6.3%)	1468 (18.2%)
Other	1 (0.1%)	4 (0.2%)	3 (0.1%)	1 (0.1%)	0	9 (0.1%)
Marital status						
Unmarried	21 (1.2%)	16 (0.7%)	22 (1.1%)	16 (0.9%)	0	75 (0.9%)
Married	1780 (98.6%)	2347 (99.2%)	2060 (98.7%)	1744 (99.0%)	32 (100.0%)	7963 (98.9%)
Widowed / separated	4 (0.3%)	4 (0.2%)	5 (0.2%)	2 (0.2%)	0	15 (0.1%)
Mother's occupation[2]						
Housewife	1374 (76.3%)	1828 (77.3%)	1671 (80.2%)	1411 (80.2%)	25 (78.1%)	6309 (78.5%)
Employed	427 (23.7%)	536 (22.7%)	412 (19.8%)	349 (19.8%)	7 (21.9%)	1731 (21.5%)

Father's occupation[3]						
Unemployed	8 (0.4%)	10 (0.4%)	5 (0.2%)	6 (0.3%)	0	29 (0.4%)
Unskilled worker	293 (16.4%)	429 (18.3%)	436 (21.0%)	374 (21.4%)	11 (35.5%)	1543 (19.3%)
Skilled worker	795 (44.4%)	982 (41.8%)	896 (43.2%)	746 (42.7%)	11 (35.5%)	3430 (42.9%)
Professional	400 (22.3%)	516 (22.0%)	412 (19.9%)	314 (18.0%)	7 (22.6%)	1649 (20.6%)
Business/self-employed	294 (16.4%)	411 (17.5%)	325 (15.7%)	306 (17.5%)	2 (6.5%)	1338 (16.7%)
Maternal education level[1]						
Up to Primary	133 (7.4%)	176 (7.4%)	166 (8.0%)	130 (7.4%)	2 (6.3%)	607 (7.5%)
Up to Ordinary level	855 (47.4%)	1154 (48.8%)	1025 (49.1%)	887 (50.3%)	18 (56.3%)	3939 (48.9%)
Up to Advanced level	569 (31.5%)	744 (31.4%)	657 (31.5%)	557 (31.6%)	10 (31.3%)	2537 (31.5%)
Diploma / Graduate	248 (13.7%)	293 (12.4%)	238 (11.4%)	188 (10.7%)	2 (6.3%)	969 (12.0%)
Father's education level[4]						
Up to Primary	160 (8.9%)	209 (8.9%)	202 (9.7%)	191 (10.9%)	3 (9.4%)	765 (9.6%)
Up to Ordinary level	938 (52.4%)	1259 (53.5%)	1146 (55.2%)	928 (52.9%)	17 (53.1%)	4288 (53.6%)
Up to Advanced level	526 (29.4%)	706 (30.0%)	567 (27.3%)	505 (28.8%)	9 (28.1%)	2313 (28.9%)
Diploma / Graduate	167 (9.3%)	179 (7.6%)	162 (7.8%)	129 (7.4%)	3 (9.4%)	640 (8.0%)
Monthly family income (LKR)[5]						
≤25000	134 (7.6%)	195 (8.4%)	180 (8.8%)	136 (8.0%)	2 (6.5%)	647 (8.3%)
25001-50000	847 (48.2%)	1165 (50.4%)	1079 (52.8%)	918 (54.2%)	20 (64.5%)	4029 (51.4%)
50001-100000	590 (33.5%)	731 (31.6%)	622 (30.5%)	499 (29.5%)	5 (16.1%)	2447 (31.2%)
>100000	188 (10.7%)	221 (9.6%)	161 (7.9%)	141 (8.3%)	4 (12.9%)	715 (9.1%)

Missing data: [1]1 subject, [2]13 subjects, [3]64 subjects, [4]47 subjects, [5]215 subjects

Supplemental Table 2 – Delivery characteristics at different gestations of mothers with or without medical or obstetric complications

	Pregnancies with at least one medical or obstetric complication					Uncomplicated pregnancies				
Mode of Delivery	***37-week gestation (N=731)***	***38-week gestation (N=670)***	***39-week gestation (N=336)***	***40-week gestation (N=179)***	***41-week gestation (N=4)***	***37-week gestation (N=1074)***	***38-week gestation (N=1697)***	***≥39-week gestation (N=1750)***	***40-week gestation (N=1583)***	***41-week gestation (N=28)***
Vaginal delivery (VD)	272 (37.2%)	346 (51.6%)	215 (64.0%)	120 (67.0%)	3 (75.0%)	428 (39.9%)	904 (53.3%)	1360 (77.7%)	1170 (73.9%)	16 (57.1%)
Spontaneous VD [1]	101 (37.3%)	138 (40.5%)	118 (56.2%)	75 (64.1%)	1 (33.3%)	322 (77.0%)	713 (80.7%)	1063 (79.7%)	712 (62.1%)	5 (31.3%)
Induced VD [1]	170 (62.7%)	203 (59.5%)	92 (43.8%)	42 (35.9%)	2 (66.7%)	96 (23.0%)	170 (19.3%)	271 (20.3%)	434 (37.9%)	11 (68.8%)
Caesarean section (CS)	459 (62.8%)	324 (48.4%)	121 (36.0%)	59 (33.0%)	1 (25.0%)	646 (60.1%)	793 (46.7%)	390 (22.3%)	413 (26.1%)	12 (42.9%)
Elective CS	302 (65.8%)	178 (54.9%)	35 (28.9%)	13 (22.0%)	0	481 (74.5%)	559 (70.5%)	92 (23.6%)	53 (12.8%)	3 (25.0%)
Emergency CS	157 (34.2%)	146 (45.1%)	86 (71.1%)	46 (78.0%)	1 (100.0%)	165 (25.5%)	234 (29.5%)	298 (76.4%)	360 (87.2%)	9 (75.0%)

Missing data: [1]95 subjects

Supplemental Table 3 – Indications for elective and emergency caesarean sections different gestations

	Elective CS					***Emergency CS***				
**Indication**	***37-week gestation (N=783)***	***38-week gestation (N=737)***	***39-week gestation (N=127)***	***40-week gestation (N=66)***	***41-week gestation (N=3)***	***37-week gestation (N=322)***	***38-week gestation (N=380)***	***39-week gestation (N=384)***	***40-week gestation (N=406)***	***41-week gestation (N=10)***
Cord prolapse/presentation	0	0	0	0	0	13 (4.0%)	3 (0.8%)	5 (1.3%)	7 (1.7%)	0
Failed instrumental delivery	0	0	0	0	0	0	0	4 (1.0%)	4 (1.0%)	0
Fetal distress (abnormal CTG, meconium-stained liquor, reduced fetal movements)	0	1 (0.1%)	2 (1.6%)	0	0	102 (31.7%)	172 (45.3%)	199 (51.8%)	230 (56.7%)	6 (60.0%)
Chorioamnionitis	0	0	0	0	0	3 (0.9%)	8 (2.1%)	7 (1.8%)	15 (3.7%)	0
Lack of progression/ obstructed labour	3 (0.4%)	0	0	3 (4.5%)	0	53 (16.5%)	85 (22.4%)	89 (23.2%)	90 (22.2%)	4 (40.0%)
Cephalic mal-attitudes/ malposition (brow/ face presentation/ hand prolapse/ malposition)	0	0	0	0	0	2 (0.6%)	3 (0.8%)	5 (1.3%)	1 (0.2%)	0
Malpresentations other than breech	6 (0.8%)	4 (0.5%)	4 (3.1%)	0	0	0	2 (0.5%)	1 (0.3%)	1 (0.2%)	0
Antepartum haemorrhage	0	1 (0.1%)	0	0	0	3 (0.9%)	1 (0.3%)	2 (0.5%)	1 (0.2%)	0
Dribbling and unfavourable cervix/ pre-labour rupture of membranes	3 (0.4%)	10 (1.4%)	2 (1.6%)	5 (7.6%)	0	13 (4.0%)	14 (3.7%)	8 (2.1%)	4 (1.0%)	0
Structural or pathological anomalies of the genital tract and pelvis, including active genital infections	11 (1.4%)	7 (0.9%)	2 (1.6%)	2 (3.0%)	0	0	1 (0.3%)	1 (0.3%)	2 (0.5%)	0
Placenta previa or low-lying placenta with or without other complications	18 (2.3%)	5 (0.7%)	0	1 (1.5%)	0	5 (1.6%)	3 (0.8%)	1 (0.3%)	0	0

IUGR and its complications	35 (4.5%)	13 (1.8%)	2 (1.6%)	2 (3.0%)	0	5 (1.6%)	3 (0.8%)	5 (1.3%)	0	0
Past caesarean section with or without obstetric or fetal complications (includes breech, preeclampsia, failed vaginal birth after caesarean)	447 (57.1%)	472 (64.0%)	62 (48.8%)	28 (42.4%)	3 (100.0%)	64 (19.6%)	43 (11.3%)	10 (2.6%)	7 (1.7%)	0
Two or more past caesarean sections	62 (7.9%)	34 (4.6%)	3 (2.4%)	1 (1.5%)	0	7(2.2%)	3 (0.8%)	2 (0.5%)	0	0
Adverse outcomes in previous pregnancies (bad obstetric history, past intrauterine death, miscarriage and recurrent miscarriage)	18 (2.3%)	4 (0.5%)	2 (1.6%)	1 (1.5%)	0	0	0	1 (0.3%)	0	0
Advanced maternal age	11 (1.4%)	10 (1.4%)	3 (2.4%)	1 (1.5%)	0	1 (0.3%)	0	0	0	0
Breech	45 (5.7%)	52 (7.1%)	8 (6.3%)	2 (3.0%)	0	14 (4.3%)	8 (2.1%)	4 (1.0%)	0	0
Hypertensive disorder of pregnancy and its complications	8 (1.0%)	7 (0.9%)	0	0	0	14 (4.3%)	5 (1.3%)	5 (1.3%)	3 (0.7%)	0
Medical conditions other than diabetes or hypertension (cardiac disease, epilepsy, immune thrombocytopenia, cholestasis, sepsis, etc.)	11 (1.4%)	12 (1.6%)	1 (0.8%)	0	0	1 (0.3%)	3 (0.8%)	3 (0.8%)	1 (0.2%)	0
Diagnosed or suspected cephalopelvic disproportion	10 (1.3%)	25 (3.4%)	17 (13.4%)	8 (12.1%)	0	3 (0.9%)	2 (0.5%)	5 (1.3%)	7 (1.7%)	0
Hyperglycaemia in pregnancy and its complications	14 (1.8%)	7 (0.9%)	2 (1.6%)	1 (1.5%)	0	3 (0.9%)	0	1 (0.3%)	0	0
Fetal anomalies	7 (0.9%)	0	0	0	0	0	0	0	0	0
Failed induction	3 (0.4%)	3 (0.4%)	4 (3.1%)	2 (3.0%)	0	10 (3.1%)	18 (4.7%)	20 (5.2%)	21 (5.2%)	0

History of myomectomy, uterovaginal surgery, or instrumentation	8 (1.0%)	4 (0.5%)	0	0	0	0	0	0	0	0
History of primary or secondary subfertility	16 (2.0%)	15 (2.0%)	3 (2.4%)	1 (1.5%)	0	1 (0.3%)	0	0	1 (0.2%)	0
Invitro fertilisation (IVF) pregnancy	9 (1.1%)	1 (0.1%)	0	0	0	2 (0.6%)	0	0	0	0
Caesarean section indicated due to other concurrent surgical requirements (e.g., LRT)	5 (0.6%)	8 (1.1%)	0	0	0	0	0	0	0	0
Suspected placenta accreta spectrum	3 (0.4%)	0	0	0	0	0	0	1 (0.3%)	0	0
Past dates	1 (0.1%)	0	0	5 (7.6%)	0	0	0	1 (0.3%)	0	0
Maternal request	17 (2.2%)	24 (3.2%)	5 (3.9%)	2 (3.0%)	0	0	0	0	0	0
Miscellaneous	1 (0.1%)	2 (0.3%)	0	1 (1.5%)	0	2 (0.6%)	1 (0.3%)	1 (0.3%)	0	0
Indication not clearly defined	11 (1.4%)	16 (2.2%)	5 (3.9%)	0	0	1 (0.3%)	2 (0.5%)	4 (1.0%)	10 (2.5%)	0

Supplemental Table 4 – Neonatal outcomes within first 24 hours of life of neonates born at different gestations

Neonatal outcome	***Frequency (%) at different gestation categories***					***Adjusted Odds Ratios (AOR) and 95%CI***		
	37-week gestation (N=1805)	***38-week gestation (N=2367)***	***39-week gestation (N=2087)***	***40-week gestation (N=1762)***	***41-week gestation (N=32)***	***AOR & 95%CI (37 vs 38 weeks) ****	***AOR & 95%CI and (38 vs 39 weeks) ****	***AOR & 95%CI and (39 vs 40 weeks) ****
Low birth weight (<2500g)[1]	529 (29.4%)	359 (15.2%)	154 (7.4%)	80 (4.5%)	0	AOR: 2.54, CI: 2.17-2.97	AOR: 2.47, CI: 2.02-3.03	AOR: 1.76, CI: 1.33-2.33
Small for gestational age[2]	338 (18.8%)	410 (17.3%)	379 (18.2%)	424 (24.1%)	14 (43.8%)	AOR:1.18, CI: 1.00-1.39	AOR: 1.03, CI: 0.88-1.20	AOR: 0.72, CI: 0.61-0.84
5-minute APGAR <8[3]	20 (1.1%)	9 (0.4%)	12 (0.6%)	20 (1.1%)	0	AOR: 3.04, CI: 1.36-6.76	AOR: 0.58, CI: 0.24-1.41	AOR: 0.47 CI: 0.23-0.99
Resuscitation at birth	97 (5.4%)	76 (3.1%)	84 (4.0%)	74 (4.2%)	2 (6.3%)	AOR: 1.74, CI: 1.27-2.38	AOR: 0.80, CI: 0.06-1.11	AOR: 0.95, CI: 0.68-1.31
Death within first 24 hours of life	2 (0.11%)	1 (0.042%)	1 (0.047%)	1 (0.056%)	0	AOR: 3.01, CI: 0.27-33.45	AOR: 1.02, CI: 0.06-17.07	AOR: 0.96, CI: 0.06-15.53
Admitted to the NICU	60 (3.3%)	47 (2.0%)	42 (2.0%)	50 (2.8%)	2 (6.3%)	AOR: 1.62, CI: 1.09-2.41	AOR: 0.90, CI: 0.59-1.39	AOR: 0.71, CI: 0.47-1.09

Missing data: [1]4 subjects, [2]5 subjects,[3]1 subjects

**AOR- Odds ratios adjusted for maternal age, parity, maternal medical and obstetric complications in logistic regression*

Supplemental Table 5 – Neonatal outcomes within first 24 hours of life of neonates delivered by elective caesarean section at different gestations

Neonatal outcome	***Frequency (%) at different gestation categories***					***Adjusted Odds Ratios (AOR) and 95%CI***		
	37-week gestation (N=781)	***38-week gestation (N=737)***	***39-week gestation (N=127)***	***40-week gestation (N=66)***	***41-week gestation (N=3)***	***AOR & 95%CI (37 vs 38 weeks) ****	***AOR & 95%CI and (38 vs 39 weeks) ****	***AOR & 95%CI and (39 vs 40 weeks) ****
Low birth weight (<2500g) [1]	186 (23.8%)	105 (14.2%)	12 (9.4%)	5 (7.6%)	0	AOR: 2.03, CI: 1.54-2.66	AOR: 1.63, CI: 0.86-3.10	AOR: 1.16, CI: 0.37-3.65
Small for gestational age [2]	121 (15.5%)	119 (16.2%)	23 (18.1%)	18 (27.3%)	2 (66.7%)	AOR:0.99, CI: 0.75-1.32	AOR:0.91, CI: 0.55-1.50	AOR:0.57, CI: 0.27-1.24
5-minute APGAR <8	5 (0.6%)	2 (0.3%)	1 (0.8%)	0	0	AOR: 2.37, CI: 0.45-12.44	AOR: 3.72, CI: 0.02-3.39	-
Resuscitation at birth	48 (6.1%)	18 (2.4%)	3 (2.4%)	3 (4.5%)	0	AOR: 2.64, CI: 1.50-4.64	AOR: 1.063, CI: 0.30-3.74	AOR: 0.58 CI: 0.11-3.05
Death within 1 hour of birth	2 (0.3%)	1 (0.1%)	0	0	0	AOR: 2.29, CI: 0.20-25.50	-	-
Admitted to the NICU	32 (4.1%)	13 (1.8%)	5 (3.9%)	1 (1.5%)	0	AOR: 2.23, CI: 1.14-4.36	AOR: 0.47, CI: 0.16-1.41	AOR: 2.54, CI: 0.28-22.92

Missing data: [1]2 subjects, [2]3 subjects

**AOR- Odds ratios adjusted for maternal age, parity, maternal medical and obstetric complications in logistic regression*

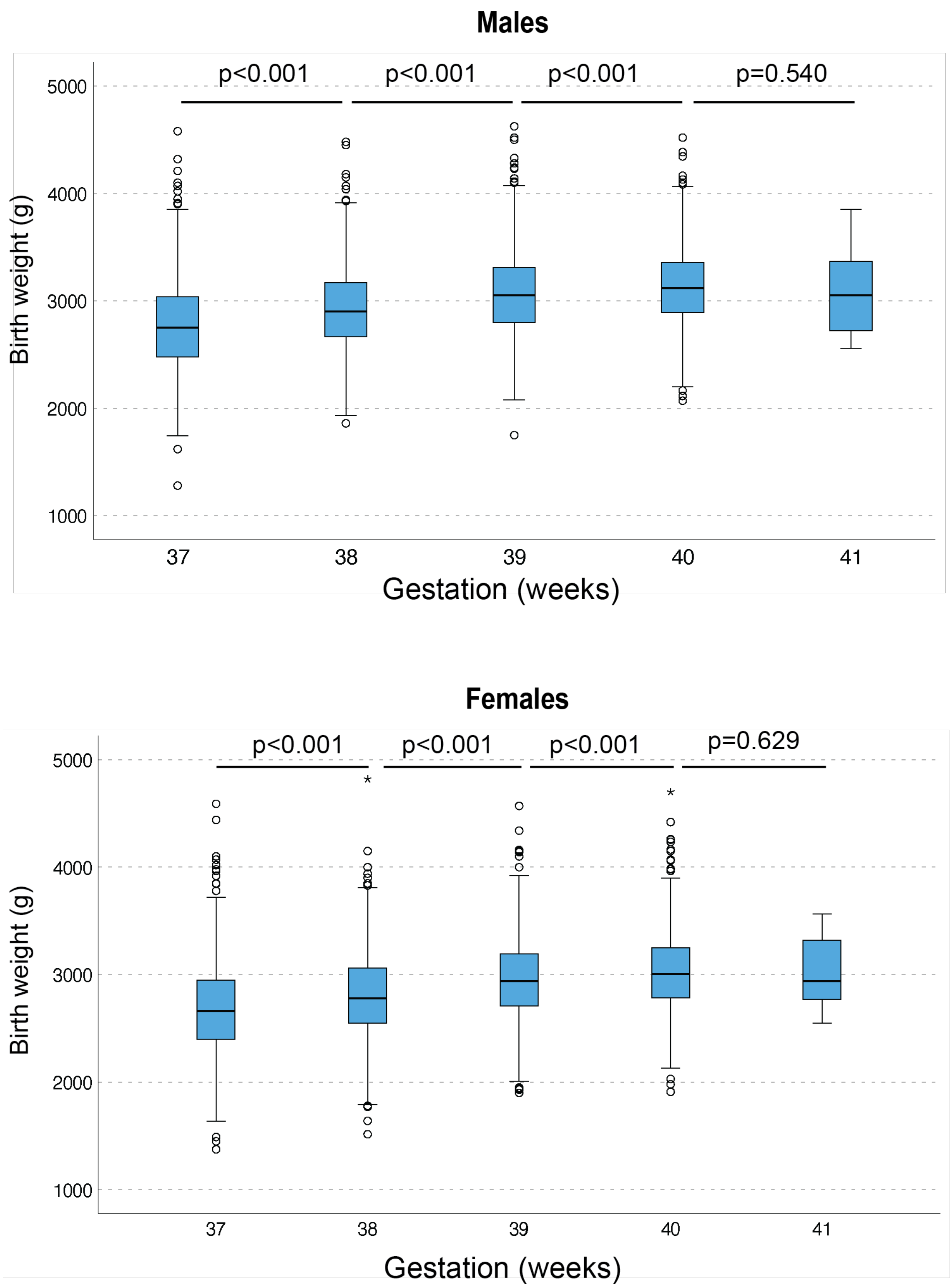

Supplemental Figure 1 – Median birth weights of male and female neonates born at different gestations at term. *Box plots show interquartile range; the middle horizontal line represents the median, error bars represent 95% confidence interval, and dots represent outliers.*
